# Development of a Digital-Based Instrument to Assess Perceived Motor Competence in Children: Face Validity, Test-Retest Reliability, and Internal Consistency

**DOI:** 10.3390/sports5030048

**Published:** 2017-07-04

**Authors:** Leah E. Robinson, Kara K. Palmer

**Affiliations:** 1 Child Movement, Activity, and Developmental Health Laboratory; School of Kinesiology, University of Michigan, Ann Arbor, MI 48109, USA; palmerka@umich.edu; 2 Center for Human Growth and Development, University of Michigan, Ann Arbor, MI 48109, USA

**Keywords:** perceived competence, motor skills, movement, children

## Abstract

Assessing children’s perceptions of their movement abilities (i.e., perceived competence) is traditionally done using picture scales—Pictorial Scale of Perceived Competence and Acceptance for Young Children or Pictorial Scale of Perceived Movement Skill Competence. Pictures fail to capture the temporal components of movement. To address this limitation, we created a digital-based instrument to assess perceived motor competence: the Digital Scale of Perceived Motor Competence. The purpose of this study was to determine the validity, reliability, and internal consistency of the Digital-based Scale of Perceived Motor Skill Competence. The Digital-based Scale of Perceived Motor Skill Competence is based on the twelve fundamental motor skills from the Test of Gross Motor Development-2nd Edition with a similar layout and item structure as the Pictorial Scale of Perceived Movement Skill Competence. Face Validity of the instrument was examined in Phase I (*n* = 56; *M*_age_ = 8.6 ± 0.7 years, 26 girls). Test-retest reliability and internal consistency were assessed in Phase II (*n* = 54, *M*_age_ = 8.7 years ± 0.5 years, 26 girls). Intra-class correlations (ICC) and Cronbach’s alpha were conducted to determine test-retest reliability and internal consistency for all twelve skills along with locomotor and object control subscales. The Digital Scale of Perceived Motor Competence demonstrates excellent test-retest reliability (ICC = 0.83, total; ICC = 0.77, locomotor; ICC = 0.79, object control) and acceptable/good internal consistency (α = 0.62, total; α = 0.57, locomotor; α = 0.49, object control). Findings provide evidence of the reliability of the three level digital-based instrument of perceived motor competence for older children.

## 1. Introduction

Children’s perceptions towards their movement ability, or perceived motor competence, is an important contributor of physical activity participation and aids in establishing a positive health trajectory [1]. A systematic review by Babic et al. [2] found that, compared to any other measure of self-concept, perceived motor competence exhibits the strongest relationship with physical activity behaviors in children and adolescents. The developmental path of this relationship is not well-established, but research supports the relationships among physical activity, perceived motor competence, and actual motor competence are dependent on age [2,3]. Specifically, young children (3–7 years) are unable to accurately perceive their motor competence and exhibit inflated perceptions of their actual abilities that drives engagement in motor activities resulting in the development of motor skills [3]. Older children (7–16 years) are able to more accurately assess their abilities resulting in stronger relationships among perceived motor competence, actual motor competence, and physical activity [3]. In other words, how well you think you move, may mediate the relationship between your actual movement abilities and physical activity engagement.

The experimental evidence examining the mediating effects of perceived competence has mixed findings. Longitudinal work by Barnett and colleagues [4] found that perceived sports competence- how you think you perform in sport context, mediates the relationship between actual motor competence in childhood (10.1 years) and self-reported physical activity engagement later during adolescence (16.4 years). However, work from others does not support the mediating effects of perceived competence [5]. Crane et al. [5] found that perceived physical competence measured using the Pictorial Scale of Perceived Competence and Social Acceptance for Young Children did not mediate the relationship between motor competence and physical activity engagement in kindergarteners (5–6 years). These studies differ in several key ways—sample age (i.e., adolescents vs. kindergartners), scales to assess perceived competence (i.e., Physical Self-Perception Profile vs. Pictorial Scale of Perceived Competence and Social Acceptance for Young Children), domain of perceived competence assessed (i.e., sports vs. motor), and length of time to follow up. Hence, more work is needed to better understand how perceived competence is related to actual motor competence and physical activity across childhood.

One particular limitation to the existing literature relating to perceived competence are the current assessments designed to measure this construct. For the past 30 years, the physical competence subscale of the Pictorial Scale of Perceived Competence and Social Acceptance of Young Children [6] has been the most used assessments to examine perceived motor competence. To complete this assessment [6], young children are presented with two static pictures: one of a highly skilled child and one of a less skilled child. Children are asked to look at the two pictures while listening to an administrator. Administrators verbally describe that one picture shows a child who is good at the skill whereas the other picture shows a child who isn’t good at the skill. Children listen to the descriptions and are prompted to point to the picture that looks the most like them. After making this choice, children are again prompted to choose to what extent they can perform the skill shown. This response results in a quantitative score between 1–4 with 4 representing the most skilled and 1 representing the least skilled. This assessment includes four subscales that measure individual constructs of perceived competence: physical, cognitive, social acceptance, and maternal acceptance. Each subscale includes 6 questions/skills that vary according to the child age. The physical subtest is commonly used to measure perceived motor competence and includes the skills swinging, climbing, tying shoes, running, skipping, and hopping for preschoolers and kindergartners and swinging, climbing, bouncing a ball, running, skipping, and using a jump rope for first and second graders. Despite the knowledge gained from this assessment [5,7,8,9,10], using it to measure perceived motor competence imposes several limitations. First, this assessment does not align with the current assessments of motor competence. Second, the assessment provides a static, pictorial description of a multi-stage, coordinated movement.

Recent work has identified additional limitations to the Pictorial Scale of Perceived Competence and Social Acceptance of Young Children [6]. Barnett and colleagues [11] addressed the first limitation with the creation and validation of the Pictorial Scale of Perceived Movement Skill Competence This scale includes eighteen movement skills: six play skills and twelve fundamental motor skills. The six play skills are common leisure time activities that Australian children participate in: swimming, boogie boarding, bicycle riding, rope climbing, roller skating, and riding a scooter and the twelve fundamental motor skills are based off the Test of Gross Motor Development-2nd Edition (TGMD-2). The TGMD-2 is a widely used, reliable and valid assessment of motor skill competence in children ages 3–10 years [12]. The skills included in this assessment are divided into locomotor skills (run, gallop, hop, leap, jump, slide) and object control skills (throw, catch, kick, dribble, roll, two-handed strike). Using motor tasks that clearly align with common assessments of actual motor skill competence provides a better understanding of how children’s self-perceptions align with their abilities. Emerging literature on Pictorial Scale of Perceived Competence and Social Acceptance of Young Children shows that it is a valid and reliable measure [11] with strong construct validity [13]. However, reliability differences exist between an Australian sample and a sample of racially diverse children from a low socioeconomic area of the United States [14].

Although the Pictorial Scale of Perceived Competence and Social Acceptance of Young Children and Pictorial Scale of Perceived Movement Skill Competence made great contributions to the study of perceived motor competence, these measures are limited due to the continued use of static pictures to depict movement. Movement is a dynamic process that happens in four-dimensions—height, depth, width and time. Pictorial representations of movement are limited. At best a picture can only portray a three-dimensional representation (height, depth, width) of a single instant in a sequenced and coordinated action, but a motor skill may take several seconds to fully execute. It might be difficult for an individual to understand and identify if they are able or unable to perform the skill without representing the temporal coordination of the movement in its entirety. Recent evidence supports that multimedia and video-based tools are effective for providing digital demonstrations of motor skills in school-age children [15]. In their study, Robinson et al. replaced a live skill demonstration with a digital demonstration during the administration of the TGMD-2. Results found that children’s scores on the TGMD-2 did not differ when they were given a live demonstration versus a digital demonstration. These findings support the use of digital tools in demonstrating movement in four-dimensions (i.e., height, depth, width, and time) to pediatric populations. The purpose of this study was to develop and determine the face validly, test-retest, and internal consistency of the Digital-based Scale of Perceived Motor Skill Competence of children during the early to middle childhood years.

## 2. Materials and Methods

### 2.1. Phase I: Face Validity

**Figure 1:**
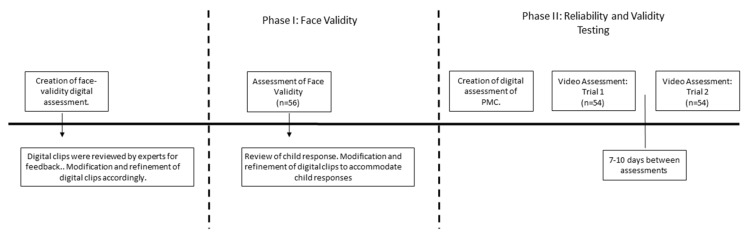
Timeline of the development and testing of the Digital-based Scale of Perceived Motor Skill Competence.

**Sample.** The sample in Phase I consisted of 56 children (*M*_age_ = 8.6 ± 0.7 years, 26 girls) from two elementary classes. The racial composition of the sample was: 75% Black, 23.2% Hispanic, and 1.2% White. A recruitment letter and consent/assent form were distributed to the parent of each child. Parental consent and child assent were obtained in accordance with the project protocol approved by the Institutional Review Board. Figure 1 provides a timeline for this study.

**Instrument Development.** The Digital-based Scale of Perceived Motor Skill Competence is a digital-based assessment that enables fundamental motor skills to be viewed in four-dimensions (i.e., height, width, depth, and time). This scale was modeled after the Pictorial Scale of Perceived Movement Skill Competence [11] and features the twelve fundamental motor skills (i.e., hop, gallop, jump, leap, run, slide, catch, dribble, kick, overhead throw roll, and strike) of the TGMD-2 [12]. Skilled and unskilled performances of the twelve skills were video recorded and edited into short, 3–6 s, digital clips. The lead author (L.E.R.) served as the model for all clips due to a majority of subjects being from similar racial backgrounds (i.e., African American) and a long history of working in this school. The skilled and unskilled performances aligned with common mature (skilled) and immature (unskilled) executions of skill completion. For example, the clip demonstrating the immature throw the showed the model completing a “shot put” motion and propelled the ball by leaning back and then pushing the ball forward. The clip with the mature throw showed the model performing skilled throwing motion with a contralateral step and weight transfer, wind up, shoulder and hip rotations, and a follow through. The clip of an immature jump showed the model taking off and landing with the right and left foot leaving and contacting the ground at different times. Further, the model kept their arms at their sides during the immature jump. The clip of the mature jump showed the model’s feet leaving and contacting the ground simultaneously as well as included preparatory arm movement, arm extension above the head, and arms were thrust down during landing. All clips were reviewed by two motor development experts external to the study to ensure that the motor skill demonstrations aligned with skilled and unskilled performances.

**Figure 2:**
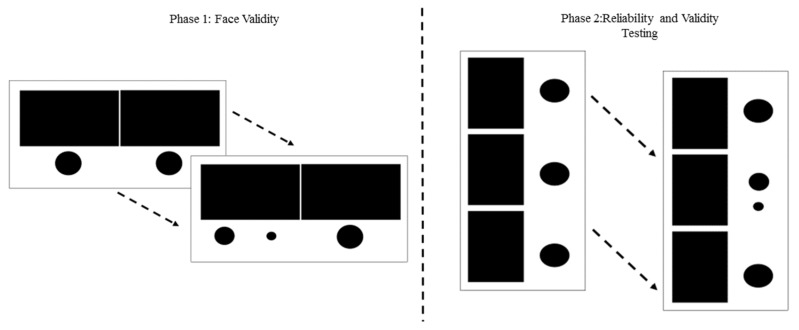
Visual representation of how the videos were presented for both Phase I and Phase II.

**Procedures.** The purpose of Phase I was to determine if children could: (A) discriminate between the skilled and unskilled motor skill performances, (B) verbally describe the difference between the skilled and unskilled performances, and (C) identify any additional factors regarding motor skill performance on the digital clips that would alter responses. For Phase I, children sat down one-on-one with a member of the research team and watched the two motor skill performances (i.e., skilled and unskilled) on a tablet screen (11.5 inch × 7.93 inch × 0.36 inch; see Figure 2 for example of skill presentation) for each of the twelve fundamental motor skills. All skills were counterbalanced between object control and locomotor skills. After watching both clips, the child would be prompted to answer the following questions: (1) which performance do you think looks like you? (2) of the two performances which is the good example and which is the not so good? and (3) why did you choose this one as good and that one as not so good? On average, it took between 12–15 min for each child to complete Phase I: Face Validity. Percentage of correct identification were examined to determine face validity.

### 2.2. Phase II: Reliability and Validity Testing

**Sample.** Fifty-four children (*M*_age_ = 8.7 years ± 0.5 years, 26 girls) served as participants for Phase II. The racial composition of the sample was: 77% Black, 21.2% Hispanic, 1.8% White. A recruitment letter and consent/assent form were distributed to the parent of each child. Parental consent and child assent were obtained in accordance with the project protocol approved by the Institutional Review Board. Figure 1 provides a timeline for this study.

**Instrument Development**. Data gathered from Phase I: Face Validity were used to make modifications to the digital clips for Phase II. If children identified specific factors external to the performer that influenced their decision in selecting the skilled and unskilled performance, modification were made to the digital clips. When examining the children’s responses from Phase I, a unique finding began to emerge when children were prompted to compare their skill level to the skill level on the digital clip. A majority (78%) felt that they were more skillful than the unskilled demonstration but not as skillful as the skilled demonstration. For example, one student stated “[I] don’t use two hands when I dribble, but I can’t always stay in one place with the ball…I move around a lot” and another stated “when I slide I go fast and get my feet in the air but sometimes I go forward instead of only sideways.” Comments like these were very common and based on the children’s comments and feedback they felt as if their skill level fell in between the two performances (i.e., unskilled and skilled) and was not represented accordingly on the assessment.

The physical education and motor development literature supports motor skill learning and acquisition occurs across three stages or abilities [16,17]. People are not simply skilled or unskilled at movement patterns, but rather fall somewhere in between the two extremes. The three stages of fundamental movements are often labeled as: initial, elementary/immediate, and mature [16,17]. The initial stage of fundamental movement represents the child’s first goal-oriented attempts at performing fundamental skills. Movement itself is characterized by missing or improperly sequenced parts, markedly restricted or exaggerated use of the body, and poor rhythmical flow of coordination. The spatial and temporal integration of movement is poor. During the elementary/intermediate phase, entails greater control and better rhythmical coordination of fundamental movement. The synchronization of the temporal and spatial elements of movement are improved, but patterns of movement are still generally restricted or exaggerated at this stage, although better coordinated. For the last stage, mature, fundamental movement is characterized by mechanically efficient, coordinated, and controlled performances. A three level versus a two level approach might be an effective approach for assessing perceived motor competence, especially in older children. Thus, for Phase II of this study revisions to the video clips were made according to student feedback as well as a third skill level was included in the assessment and children viewed three skill performances (i.e., L1, not skilled [initial]; L2, somewhat skilled [elementary/immediate]; L3, highly skilled [mature]) for each skill. The motor development expert from Phase I of this study along with two experts reviewed all clips to ensure they aligned with the three levels of performance. Clips were presented in the exact same format as described above (see Figure 2 for example of presentation).

**Procedures.** The purpose of Phase II: Reliability and Validity Testing was to determine test-retest reliability and internal consistency of the new assessment. For completion of these measures, children sat one on one with a member of the research team and watched all three clips for each skill. No verbal description of the level of skill was provided. Children were instructed to “Watch the clips and choose the clip that looks like you”. A child touched the circle below their preferred digital recording on the screen to select a clip. The circles disappeared and were replaced with a larger circle and a smaller circle. Children were then prompted to identify to “what extent they could perform the skill?”. If a child selected the L1 performance they were prompted with, “Can you not do this skill? or sort of do this skill?,” for L2, “Are you okay at this skill? or better than okay at this skill?”, and for L3, “Are you pretty good at this skill? or really good at this skill?”. Responses were recorded and corresponded with a quantitative value between 1 and 6 with 1 representing not able to do the skill at all and 6 representing being really good at that skill. All participants completed the re-test 7–10 days later. The re-test assessment was presented in reverse order to eliminate ordering effect.

**Data Analysis.** Statistical cut points were modeled from a recently published paper on face validity and internal consistency of a perceived competence measure [14]. Cronbach’s alpha was used to determine internal consistency. Alpha levels above 60 were considered to be acceptable [18]. Intra-class correlation (ICC) was used to determine test-retest reliability. ICC’s were interpreted as <0.40 as low, 0.41–0.75 as fair to good, and >0.75 as excellent [19]. Both Cronbach’s alpha and ICC were calculated for each of two subscales (locomotor and object control) as well as the entire assessment for girls, boys and the entire sample.

## 3. Results

### 3.1. Phase I: Face Validity

Findings show that children were able to correctly identify the skilled and unskilled motor skill performances (*M* = 87.9%, range 58.6–96.6%; see Table 1 provide the percentage of skills correctly identified and correctly labeled).

Children experienced the most difficulty in identifying the good performance for kick (77.6%), hop (77.6%), and overarm throw (58.6%), so these three skills were re-recorded to eliminate any concerns. For example, children commented that on the unskilled performance of the kick “the person did not stop running” (e.g., continued the forward momentum movement) whereas on the skilled performance “the person stopped running after they contacted the ball”. The kick was then re-recorded where the person ran for the same distance in both the skilled and unskilled performance. Similar procedures were undertaken for both the hop and throw.

### 3.2. Phase II: Reliability and Validity Testing

This assessment demonstrates an acceptable overall internal consistency (α = 0.62) and excellent test-retest reliability (ICC = 0.83 [0.71–0.90, 95% CI]). Alpha levels for both the locomotor and object control subtest did not reach an acceptable level for the total sample (α = 0.49, α = 0.48; respectively). However, ICC for each subtest remained excellent (ICC = 0.77 [0.61–0.87, 95% CI]; ICC = 0.79 [0.64–0.88, 95% CI]). See Table 2 for alpha’s and ICC for each subscale according to sex.

## 4. Discussion

The study examined the face validity, test-retest reliability, and internal consistency of a digital-based instrument designed to measure perceived motor competence in young children. The digital-based scale was developed to address two major assessment limitations—the lack of alignment between motor skills and perceived physical competence assessments and the inability of current perceived physical competence measures to represent multi-stage, coordinated movement [6,11]. A digital-based scale that represents the dynamic process of movement will allow individuals to see movement in four-dimensions—height, depth, width and time while a pictorial only represents three-dimensional (i.e., height, depth, width). A picture or pictorial assessment truly limits an individual’s ability to understand the temporal coordination of a movement pattern that in turn limits their ability to understand the movement. These limitations could ultimately influence perceptions.

Results from this study show that the digital-based instrument demonstrated excellent face validity. Nearly all of the children (average = 88%) were able to correctly identify the skilled/unskilled performance and articulate key features of the movement action that classified the skill. Overall, the digital-based instrument demonstrated acceptable internal consistency (α = 0.62) and upheld excellent test-retest reliability (ICC = 0.83). The digital-based instrument is modified based on the Pictorial Scale of Perceived Movement Skill Competence which also had good internal consistency [ICC= 0.76; 11].

There are some limitations in the present study. For Phase I and Phase II, the lead investigator (LR) was the model in the digital clips. This might be seen as a limitation but the decision was made for two reasons. First, a majority of the children were from a similar racial background (i.e., Black) and the researcher had previously established rapport with the children. Previous work poses no concern using adult and/or female models to demonstrate motor skills for a digital-based multimedia tools in school-age children [15]. Work using video models for children with Autism supports that adult models are effective for teaching social language [20], social initiation [21], and expressive language [22]. Two of these studies used both a peer and adult models but no differences were seen in behavior outcomes according to video models [21,22]. Lastly, work with typically developing preschoolers shows that children may prefer an adult model to a peer model in regards to certain information [23]. One study found that children selected adult models for new food but selected peer models for information on toys [23]. Another study found that when learning new words preschoolers selected models based on perceived reliability not according to age [24]. Based on this literature, we feel using a familiar and reliable adult model to demonstrate a motor task was appropriate.

Another limitation was the absence of face validity during Phase II. Establishing face validity would provide additional feedback from the children’s perspective on the three level approach. This inclusion was not feasible due to various constraints within in the school setting (i.e., end of the academic semester, final examination, student absences due to illnesses). Concerns regarding the digital clips themselves was potentially alleviated by having the digital clips reviewed by three experts in the field prior to their use in either Phase I or Phase II. Lastly, children included in both Phase I and II were from the same school creating the potential for contamination between the samples, but there appeared to be no concerns for contamination during the data collection for Phase II. Future investigations should be aware of these limitations along with others (e.g., a larger and more diverse sample) and aim to better eliminate them to strengthen future work in this field. Further reliability testing of the digital instrument is needed in a larger sample across a range of age groups to support the generalizability.

It appears that the Pictorial Scale of Perceived Movement Skill Competence has relatively higher internal consistency compared to this Digital-based Scale of Perceived Motor Skill Competence, an alpha of 0.76 and 0.62, respectively. Both of these assessments are above the alpha of 0.60 that is considered acceptable according to Sim and Wright [18]. It should also be noted that both of these scales are higher than Harter and Pike’s reported internal consistency for perceived physical competence for older children [i.e., 1st/2nd graders; ICC= 0.53; 6]. Additionally, the internal consistency values for the Digital-based Scale of Perceived Motor Competence were deemed acceptable for use in both boys and girls [11,19]. Differences in internal consistency values for the Digital-based Scale of Perceived Motor Skill Competence might be attributed to factors stated within the limitations and to taking a three-level approach to perceived competence versus a two-level approach. As mentioned before, the model was an African American female adult, who was extremely familiar to the participants. This could have affected internal consistency but despite having an adult model, the Digital-based Scale of Perceived Motor Skill Competence demonstrated excellent test-retest reliability and acceptable/good internal consistency.

## 5. Conclusions

The Digital-based Scale of Perceived Motor Skill Competence offers a unique contribution to the perceived motor competence literature and potentially advance the motor development field. This tool addresses limitations from past work on perceived physical and motor competence in young children by aligning this assessment with fundamental motor skills and represents four-dimensional multi-stage, coordinated movement. This Digital-based Scale of Perceived Motor Skill Competence also incorporated three stages of motor skill learning commonly seen in the physical education and motor skill development literature [16,17]. Research clearly states that perceptions of movements are critically important to our current and future motor actions and behaviors [2]. The Digital-based Scale of Perceived Motor Skill Competence enables researchers to assess perceived motor competence in the dynamic process in which movement occurs.

## Figures and Tables

**Table 1:** Percentage of skills correctly identified and correctly labeled.

Object Control Skills	% Correct	Locomotor Skills	% Correct
Catch	94.8	Gallop	91.4
Dribble	87.9	Hop	77.6
Kick	77.6	Jump	96.6
Roll	91.4	Leap	93.1
Strike	96.6	Run	94.8
Throw	58.6	Slide	94.8
Total	87.5	Total	94.6

**Table 2:** Alpha levels and ICC for each subtest and total assessment.

Subtest	Sex	Alphas Test 1	ICC (LCI-UCI)
Object control	All	0.48	0.79 (0.64–0.88)
	Girls	0.55	0.80 (0.56–0.91)
	Boys	0.42	0.77 (0.50–0.89)
Locomotor	All	0.49	0.77 (0.61–0.87)
	Girls	0.68	0.79 (0.53–0.91)
	Boys	0.58	0.75 (0.46–0.88)
Total	All	0.62	0.83 (0.71–0.90)
	Girls	0.58	0.82 (0.61–0.92)
	Boys	0.64	0.83 (0.65–0.92)
