# Erratum: Robinson, L.E., et al. Development of a Digital-Based Instrument to Assess Perceived Motor Competence in Children: Face Validity, Test–Retest Reliability, and Internal Consistency. *Sports* 2017, *5*, 48

**DOI:** 10.3390/sports5040093

**Published:** 2017-12-08

**Authors:** Sports Editorial Office

**Affiliations:** MDPI AG, St. Alban-Anlage 66, 4052 Basel, Switzerland; sports@mdpi.com

The editorial team of the journal *Sports* would like to make the following corrections to the published paper [1]: The references from 1 to 12 should be modified as follows:

## Original References

1. Harter, S.; Pike, R. The pictorial scale of perceived competence and social acceptance for young children. *Child Dev.* **1984**, *55*, 1969–1982.
2. Barnett, L.M.; Ridgers, N.D.; Zask, A.; Salmon, J. Face validity and reliability of a pictorial instrument for assessing fundamental movement skill perceived competence in young children. *J. Sci. Med. Sport* **2015**, *18*, 98–102.
3. Ulrich, D.A. *Test of Gross Motor Development-2*; Prod-Ed: Austin, TX, USA, 2000.
4. Robinson, L.E.; Stodden, D.F.; Barnett, L.M.; Lopes, V.P.; Logan, S.W.; Rodrigues, L.P.; D’Hondt, E. Motor competence and its effect on positive developmental trajectories of health. *Sports Med.* **2015**, *45*, 1273–1284.
5. Babic, M.J.; Morgan, P.J.; Plotnikoff, R.C.; Lonsdale, C.; White, R.L.; Lubans, D.R. Physical activity and physical self-concept in youth: Systematic review and meta-analysis. *Sports Med.* **2014**, *44*, 1589–1601.
6. Stodden, D.F.; Goodway, J.D.; Langendorfer, S.J.; Roberton, M.A.; Rudisill, M.E.; Garcia, C.; Garcia, L.E. A developmental perspective on the role of motor skill competence in physical activity: An emergent relationship. *Quest* **2008**, *60*, 290–306.
7. Barnett, L.M.; Morgan, P.J.; van Beurden, E.; Beard, J.R. Perceived sports competence mediates the relationship between childhood motor skill proficiency and adolescent physical activity and fitness: A longitudinal assessment. *Int. J. Behav. Nutr. Phys. Act.* **2008**, *5*, 40–52.
8. Crane, J.R.; Naylor, P.J.; Cook, R.; Temple, V.A. Do perceptions of competence mediate the relationship between fundamental motor skill proficiency and physical activity levels of children in kindergarten? *J. Phys. Act. Health* **2015**, *12*, 954–961.
9. LeGear, M.; Greyling, L.; Sloan, E.; Bell, R.I.; Williams, B.-L.; Naylor, P.-J.; Temple, V.A. A window of opportunity? Motor skills and perceptions of competence of children in kindergarten. *Int. J. Behav. Nutr. Phys. Act.* **2012**, *9*, 29–34.
10. Robinson, L.E.; Rudisill, M.E.; Goodway, J.D. Instructional climates in preschool children who are at-risk. Part ii: Perceived physical competence. *Res. Q. Exerc. Sport* **2009**, *80*, 543–551.
11. Robinson, L.E. Effect of a mastery climate motor program on object control skills and perceived physical competence in preschoolers. *Res. Q. Exerc. Sport* **2011**, *82*, 355–359.
12. Robinson, L.E. The relationship between perceived physical competence and fundamental motor skills in preschool children. *Child Care Health Dev*. **2011**, *37*, 589–596.

## Correct References

1. Robinson, L.E.; Stodden, D.F.; Barnett, L.M.; Lopes, V.P.; Logan, S.W.; Rodrigues, L.P.; D’Hondt, E. Motor competence and its effect on positive developmental trajectories of health. *Sports Med.* **2015**, *45*, 1273–1284.
2. Babic, M.J.; Morgan, P.J.; Plotnikoff, R.C.; Lonsdale, C.; White, R.L.; Lubans, D.R. Physical activity and physical self-concept in youth: Systematic review and meta-analysis. *Sports Med.* **2014**, *44*, 1589–1601.
3. Stodden, D.F.; Goodway, J.D.; Langendorfer, S.J.; Roberton, M.A.; Rudisill, M.E.; Garcia, C.; Garcia, L.E. A developmental perspective on the role of motor skill competence in physical activity: An emergent relationship. *Quest* **2008**, *60*, 290–306.
4. Barnett, L.M.; Morgan, P.J.; van Beurden, E.; Beard, J.R. Perceived sports competence mediates the relationship between childhood motor skill proficiency and adolescent physical activity and fitness: A longitudinal assessment. *Int. J. Behav. Nutr. Phys. Act.* **2008**, *5*, 40–52.
5. Crane, J.R.; Naylor, P.J.; Cook, R.; Temple, V.A. Do perceptions of competence mediate the relationship between fundamental motor skill proficiency and physical activity levels of children in kindergarten? *J. Phys. Act. Health* **2015**, *12*, 954–961.
6. Harter, S.; Pike, R. The pictorial scale of perceived competence and social acceptance for young children. *Child Dev.* **1984**, *55*, 1969–1982.
7. LeGear, M.; Greyling, L.; Sloan, E.; Bell, R.I.; Williams, B.-L.; Naylor, P.-J.; Temple, V.A. A window of opportunity? Motor skills and perceptions of competence of children in kindergarten. *Int. J. Behav. Nutr. Phys. Act.* **2012**, *9*, 29–34.
8. Robinson, L.E.; Rudisill, M.E.; Goodway, J.D. Instructional climates in preschool children who are at-risk. Part ii: Perceived physical competence. *Res. Q. Exerc. Sport* **2009**, *80*, 543–551.
9. Robinson, L.E. Effect of a mastery climate motor program on object control skills and perceived physical competence in preschoolers. *Res. Q. Exerc. Sport* **2011**, *82*, 355–359.
10. Robinson, L.E. The relationship between perceived physical competence and fundamental motor skills in preschool children. *Child Care Health Dev*. **2011**, *37*, 589–596.
11. Barnett, L.M.; Ridgers, N.D.; Zask, A.; Salmon, J. Face validity and reliability of a pictorial instrument for assessing fundamental movement skill perceived competence in young children. *J. Sci. Med. Sport* **2015**, *18*, 98–102.
12. Ulrich, D.A. *Test of Gross Motor Development-2*; Prod-Ed: Austin, TX, USA, 2000.

We apologize for any inconvenience caused to the readers or authors by this error. The change does not affect the scientific results. The article will be updated and the original will remain on the article webpage.

## References

[1] Robinson L.E., Palmer K.K. (2017). Development of a Digital-Based Instrument to Assess Perceived Motor Competence in Children: Face Validity, Test-Retest Reliability, and Internal Consistency. Sports.

